# Low-Supervision Asynchronously Monitored Home Rehabilitation for Adhesive Capsulitis: A Pilot Randomized Study

**DOI:** 10.3390/life16091551

**Published:** 2026-09-16

**Authors:** Hsin-Wei Lin, Chia-Chen Hu, Hui-Min Yen, Jiunn-Horng Kang

**Affiliations:** 1 Department of Medical Education, Taipei Medical University Hospital, Taipei 110301, Taiwan; 2 School of Medicine, College of Medicine, Kaohsiung Medical University, Kaohsiung 807378, Taiwan; 3 Department of Physical Medicine and Rehabilitation, Taipei Medical University Hospital, Taipei 110301, Taiwan; 4 Division of Physical Therapy, Department of Physical Medicine and Rehabilitation, Taipei Medical University Hospital, Taipei 110301, Taiwan; 5 Graduate Institute of Nanomedicine and Medical Engineering, College of Biomedical Engineering, Taipei Medical University, Taipei 110301, Taiwan; 6 Department of Physical Medicine and Rehabilitation, School of Medicine, College of Medicine, Taipei Medical University, Taipei 110301, Taiwan

**Keywords:** adhesive capsulitis, frozen shoulder, digital rehabilitation, home exercise, telerehabilitation, gamification, asynchronous monitoring, physical therapy

## Abstract

Background: Adhesive capsulitis often requires prolonged rehabilitation, and home exercise performed without supervision is difficult to document objectively. This pilot randomized trial evaluated the preliminary clinical effects and system-recorded engagement of a low-supervision, asynchronously monitored home rehabilitation system used as an adjunct to conventional outpatient rehabilitation. Methods: Thirty adults with adhesive capsulitis were randomly assigned 1:1 at a university-affiliated outpatient rehabilitation department. Both groups received conventional outpatient rehabilitation twice weekly for 4 weeks; the intervention group additionally used a gamified, smartphone-based home program (HappyGoGo) with asynchronous system monitoring, and the control group received standard home exercise instructions. Because the outcome hierarchy was assigned at retrospective registration, after data collection and analysis had been completed, all outcomes are reported as exploratory. Fourteen clinical outcomes were analyzed by analysis of covariance adjusting for the corresponding baseline value, with Benjamini–Hochberg correction; unadjusted change scores were compared as a sensitivity analysis. Results: Substantial baseline imbalance was present, with the intervention group more restricted in range of motion and reporting higher pain. After adjustment, no outcome reached nominal significance and the 95% confidence interval included the null for all 14 outcomes. The largest estimate was for pain (adjusted mean difference −1.30 points, 95% CI −2.64 to 0.04; *p* = 0.057). In the unadjusted analysis, pain and internal rotation reached nominal significance but neither survived correction for multiple comparisons. All 30 participants completed the protocol, no adverse events were reported, and engagement was retrieved for 14 of 15 intervention participants (mean 61.0%, range 7.1–96.4%). Conclusions: This trial found no detectable between-group difference on any clinical outcome, with confidence intervals too wide to determine whether a clinically meaningful effect exists. The system could be implemented alongside routine care without adverse events, and participation could be documented automatically, though it varied widely between individuals.

## 1. Introduction

Adhesive capsulitis, commonly referred to as frozen shoulder, is a disabling shoulder disorder characterized by pain, progressive restriction of active and passive range of motion, and impaired upper-limb function [1]. Its reported prevalence has been estimated to reach up to 5.3%, and the condition is associated with substantial morbidity, including pain, sleep disturbance, anxiety, and limitations in daily activities [2]. Pathophysiological studies suggest that adhesive capsulitis involves pathological changes in the anterior shoulder capsule and related structures, with evidence implicating immune, inflammatory, and fibrotic processes [2]. Although frozen shoulder has traditionally been described as a self-limiting condition, complete spontaneous recovery should not be assumed, and persistent pain or mobility deficits may remain in some patients [3]. Several conservative and interventional treatments have been used for adhesive capsulitis, including physical therapy, manipulation under anesthesia, corticosteroid injection, hydrodilatation, and arthroscopic capsular release [4]. Effective rehabilitation for frozen shoulder often requires repeated, symptom-guided shoulder movement within tolerable limits [5,6]. However, participation in unsupervised home-based rehabilitation is difficult to verify objectively because conventional adherence measures often rely on self-report and are not consistently validated [7]. These challenges highlight the clinical rationale for technology-supported home-rehabilitation systems that can complement hospital-based therapy by standardizing exercise content and supporting repeated practice. Separately, such systems can record participation objectively, addressing a measurement problem that self-report cannot solve [8].

Digital rehabilitation and telerehabilitation have increasingly been applied to shoulder rehabilitation, reflecting a broader shift toward extending therapeutic exercise beyond face-to-face clinical settings [9,10]. Recent work in shoulder rehabilitation has explored gamified exercise platforms, remote monitoring, wearable systems, and camera-based approaches to support home-based training and provide clinically relevant information on rehabilitation progress [11,12]. In parallel, advances in markerless camera-based motion tracking have made rehabilitation-relevant movement assessment more accessible without requiring complex laboratory equipment [13,14]. These developments are particularly relevant to frozen shoulder, a condition that often requires repeated, multidirectional home exercise and may benefit from clearer home-based guidance and objective monitoring of participation.

Digital home rehabilitation approaches have also been explored specifically for frozen shoulder and adhesive capsulitis, but the available evidence remains mixed. Smartphone application–supported self-rehabilitation has demonstrated high patient satisfaction, but evidence of superiority over conventional self-exercise in pain or range of motion remains unclear [15]. Wearable motion sensor systems have demonstrated feasibility in monitoring shoulder movement and supporting home-based exercise, with potential improvements in exercise completion and functional recovery; however, these findings remain largely at the pilot level [16]. More recently, an augmented reality (AR)/Kinect-based telerehabilitation system incorporating physician-supervised remote monitoring demonstrated clinical improvements over time in both the telerehabilitation and conventional home-exercise groups, without a significant group-by-time interaction, suggesting that the added value of technology may depend not only on hardware capabilities but also on the monitoring model and supervision intensity [17]. Together, these findings suggest that digital rehabilitation for adhesive capsulitis remains an evolving field and that different delivery models may have distinct roles depending on monitoring strategy, level of supervision, and patient characteristics. Critically, however, two distinct questions remain unaddressed in this population: first, whether a home rehabilitation system operating under low supervision and without real-time therapist interaction produces additional clinical benefit when added to conventional outpatient rehabilitation; and second, whether asynchronous monitoring can objectively document home training participation. The latter concerns technical and operational feasibility rather than comparative effectiveness, because this type of engagement can only be recorded in participants using the system.

To address these questions, we conducted a pilot randomized trial in which a gamified, motion-tracking-based home rehabilitation system was added to conventional outpatient rehabilitation and used without real-time therapist supervision over 4 weeks. The design addresses the two questions differently by necessity. For the first, the randomized comparison provides preliminary effect estimates across pain, range of motion, strength, upper-limb function, and quality of life, together with the precision of those estimates. For the second, the system’s automated logging provides a day-level record of home-training participation in the intervention group; because no equivalent record exists for participants performing conventional home exercise, this component speaks to whether participation can be captured at all, not to whether it differs between groups. Consistent with a pilot design, the trial was not sized to test superiority, and the analyses reported here are exploratory. This report aims to describe the direction and precision of preliminary effects, to document engagement and safety under low-supervision home use, and to provide the variability estimates required for sizing a confirmatory trial.

## 2. Materials and Methods

This study was a prospective, randomized, parallel-group pilot trial with a 1:1 allocation ratio, conducted at Taipei Medical University Hospital between April 2024 and July 2025. Participants were randomly assigned to either the intervention or control group using a random number table generated by the study coordinator; no stratification or blocking was applied. The personnel responsible for recruiting and screening participants had access to the allocation sequence at the time of enrollment, and allocation concealment was therefore not implemented. Due to the nature of the intervention, neither participants nor treating personnel could be blinded to group allocation. Outcome assessment was likewise unblinded, as all assessments were performed by the physical therapist who also delivered the physical therapy and the HappyGoGo system training. The absence of assessor blinding is particularly relevant for participant-reported outcomes such as the visual analog scale. No patients or members of the public were involved in the design, conduct, or reporting of this study.

The study protocol was approved by the Institutional Review Board of Taipei Medical University Hospital (approval number: N202312062) before participant enrollment. The trial was registered on ClinicalTrials.gov on 19 July 2026 (NCT07723755), approximately one year after completion of data collection. Prospective registration was omitted because the investigators did not recognize at study initiation that the trial fell within applicable registration requirements. The registry record is available at https://clinicaltrials.gov/study/NCT07723755 (accessed on 9 September 2026). The IRB-approved protocol (version dated 13 October 2023) is available from the corresponding author on request; no separate prespecified statistical analysis plan was prepared. No substantive changes were made to the study design, interventions, eligibility criteria, assessment schedule, or collected clinical outcomes after enrollment commenced. The outcome hierarchy and the statistical analyses reported here were specified only at the time of registration, after data collection and analysis had been completed; accordingly, all outcome analyses in this report are exploratory rather than confirmatory.

Consistent with its pilot design, the trial was not supported by a formal sample-size calculation for confirmatory superiority testing. A target sample size of 30 participants was selected to provide preliminary estimates of treatment effects and system use to inform future adequately powered trials.

Eligible participants were adults aged 20 years or older with a clinical diagnosis of adhesive capsulitis made by a physician board-certified in Physical Medicine and Rehabilitation, who were able to follow instructions and complete the required assessments. The IRB-approved protocol specified “clinical diagnosis of adhesive capsulitis” without further operational criteria; no quantitative thresholds for range-of-motion restriction were prospectively defined, disease stage was not classified, symptom duration was not recorded, and imaging was obtained at the discretion of the treating physician rather than as an eligibility requirement. No exclusion criteria for competing shoulder disorders, such as rotator cuff pathology, calcific tendinopathy, glenohumeral osteoarthritis, or cervical radiculopathy, were prospectively specified, and their exclusion was not documented at enrolment. Table 1The baseline range-of-motion values documented that participants presented with multidirectional restriction, most pronounced in external and internal rotation.. Participants were excluded if they had cognitive impairment (e.g., dementia), severe psychiatric disorders (e.g., delirium), or any condition that prevented them from understanding or completing the study questionnaires. Individuals with any medical condition that contraindicated participation in routine exercise were also excluded. All participants provided informed consent prior to enrollment. Conventional outpatient interventions involving medical procedures (e.g., joint injection) and patient education were delivered by the recruiting physician; physical therapy and the HappyGoGo system training were delivered by a single physical therapist throughout the study.

**Figure 1:**
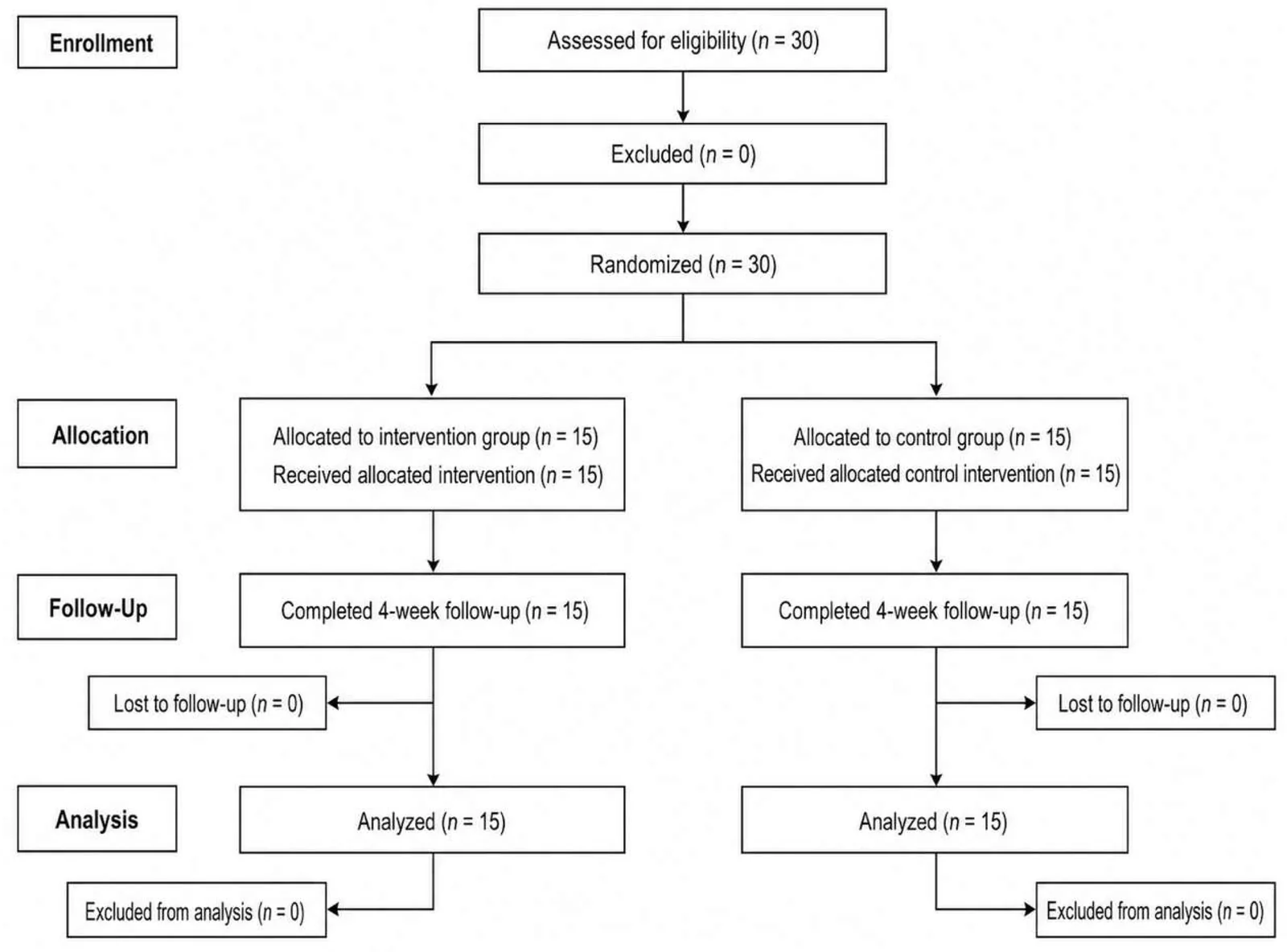
CONSORT flow diagram and intervention protocols.

Both groups received conventional outpatient rehabilitation twice weekly throughout the 4-week study period (Figure 1), comprising thermotherapy, electrotherapy, manual therapy (including stretching exercises, joint mobilization, and soft tissue massage), and therapeutic exercise. In addition, participants in the intervention group received a home-based interactive rehabilitation program using the HappyGoGo interactive rehabilitation software (LongGood MediTech Co., Ltd., Taipei, Taiwan). The system comprised two hardware platforms delivering identical training content: an in-clinic Kinect-based motion-tracking platform (Microsoft Corporation, Redmond, WA, USA) used for initial familiarization, and a smartphone-based platform (iPhone; Apple Inc., Cupertino, CA, USA) for home use, employing MediaPipe-based markerless motion tracking (Google LLC, Mountain View, CA, USA). The MediaPipe-based tracking algorithm was configured to output joint-angle data in a format consistent with the Kinect platform (25 skeletal landmarks, approximately 30 Hz sampling rate) to support comparability between in-clinic and home-based sessions. At baseline, participants were instructed in operating the system on the in-clinic platform, including device setup, screen connection, and exercise program execution; after familiarization, they were provided with a mobile device with the installed software for home use.

The home-based program consisted of daily 15 min exercise sessions guided by pre-assigned training modules delivered through an internet-connected platform. The training program focused on upper-limb functional movements, including shoulder flexion and abduction, push–pull movements, internal and external rotation, and elbow movements.

**Figure 2:**
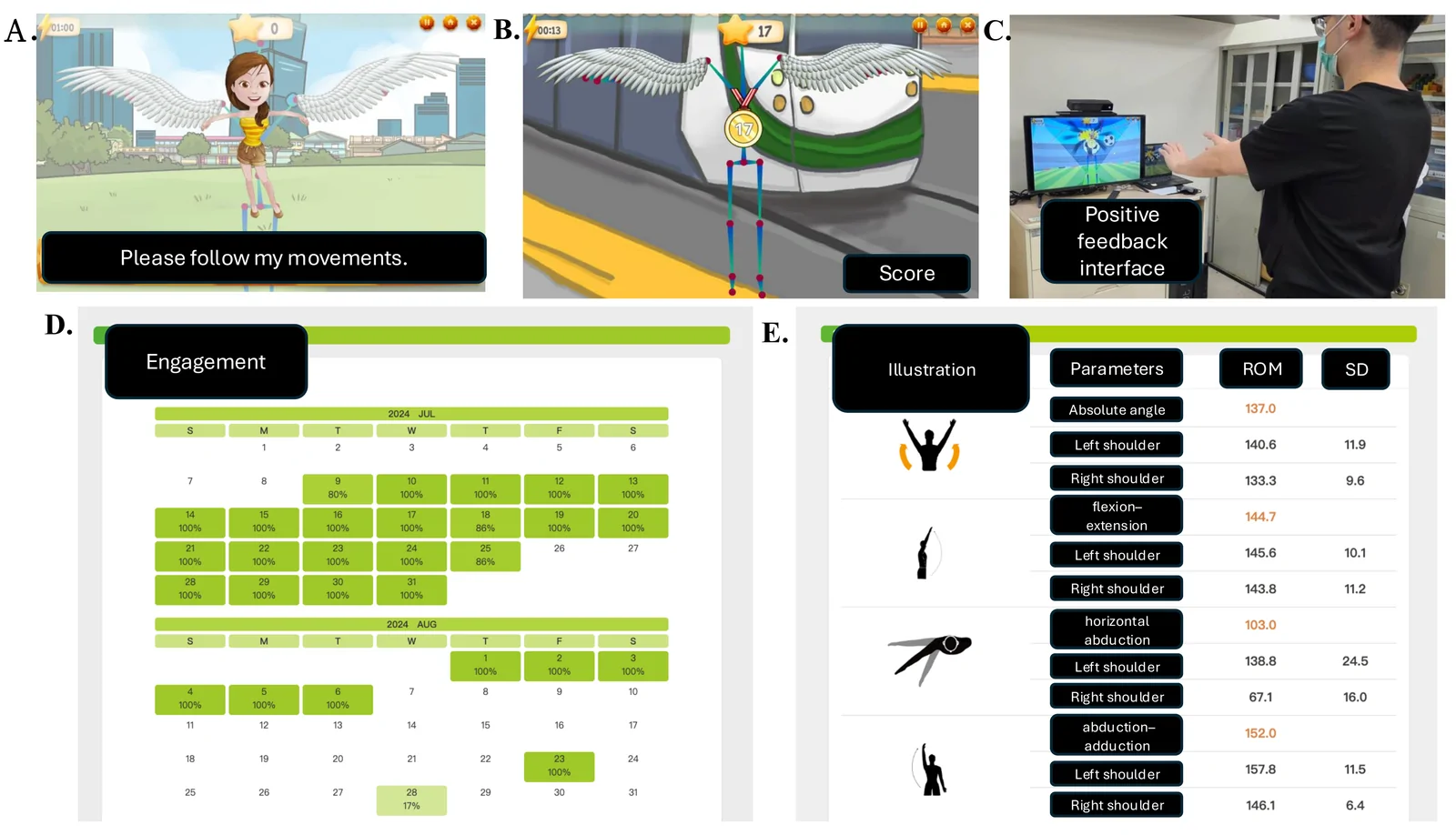
HappyGoGo interactive rehabilitation system and monitoring interface. (**A**) Guided exercise instruction interface showing the virtual coach and motion task. (**B**) Avatar position calibration and real-time scoring interface based on motion tracking. (**C**) In-clinic demonstration of the Kinect-based training platform by a research team member, illustrating real-time motion tracking and positive feedback during system familiarization. (**D**) Backend dashboard showing system-recorded engagement and daily training completion. (**E**) Joint-angle report summarizing movement parameters, range of motion, and standard deviation.

The system automatically recorded and uploaded training data to a cloud-based platform after each session. The recorded data included session completion, task achievement rates, and movement-related metrics, and were reviewed periodically for monitoring purposes (Figure 2). Participants in the control group were additionally provided with written home exercise instructions; exercise content was standardized but adjusted by the treating therapist based on individual clinical presentation and tolerance. Outcome assessments were conducted at baseline and after 4 weeks in both groups. Concomitant care, including analgesic medication use and other treatments received outside the study protocol, was not systematically monitored or restricted during the trial period.

Pain intensity was assessed using the Visual Analog Scale (VAS). Passive shoulder range of motion and muscle strength, including grip strength, were measured using a microFET3 digital dynamometer and inclinometer (Hoggan Scientific, LLC, Salt Lake City, UT, USA). Upper-limb function was assessed with the Disabilities of the Arm, Shoulder and Hand (DASH) questionnaire, and health-related quality of life with the 36-Item Short Form Health Survey (SF-36). Adverse events were assessed by actively questioning participants at each twice-weekly in-clinic rehabilitation visit throughout the 4-week intervention period. The IRB-approved protocol specified the assessment of pain, joint angle measurements, grip strength, DASH, and SF-36 but did not prospectively define a primary–secondary outcome hierarchy. In the registry record, pain and joint angle measurements were entered as primary outcome measures; DASH, grip strength, and SF-36 as secondary outcome measures; and adverse events and device compliance as other outcome measures. Multidirectional shoulder-strength measurements beyond grip strength were also collected during the study and are analyzed here as additional outcomes.

System-recorded training engagement was evaluated in the intervention group. A completed home-training day was defined as any day on which the system recorded completion of at least one exercise set within a home-based training session; days on which the application was opened without completion of an exercise set were not counted. The engagement rate was calculated as the number of such days divided by the 28 available intervention days, expressed as a percentage, and was summarized among participants for whom system records could be retrieved. Engagement was recorded only in the intervention group, as no comparable system-based record of home exercise was available for control participants, and these data are reported descriptively as a measure of system usage rather than as a clinical outcome. The exercise-completion threshold entered under Other Outcome Measures in the retrospective registry reflected a protocol-based monitoring criterion; it was not used to define engagement in this report, and no threshold-based outcome analysis was performed. Table 2 The descriptive attainment thresholds were chosen post hoc for summary purposes.

All analyses were conducted on an intention-to-treat basis. As all 30 enrolled participants completed the 4-week follow-up with no loss to follow-up, the intention-to-treat and per-protocol populations were identical.

Baseline characteristics were summarized descriptively as mean ± standard deviation or n (%). In accordance with CONSORT guidance, no significance tests were applied to baseline comparisons; standardized mean differences were computed instead, with an absolute value exceeding 0.50 taken to indicate imbalance of a magnitude warranting covariate adjustment. For continuous variables the standardized mean difference was the difference in means divided by the pooled baseline standard deviation; for dichotomous variables it was the difference in proportions divided by the pooled standard deviation of the binomial proportions, so the two are reported on a common scale.

**Figure 3:**
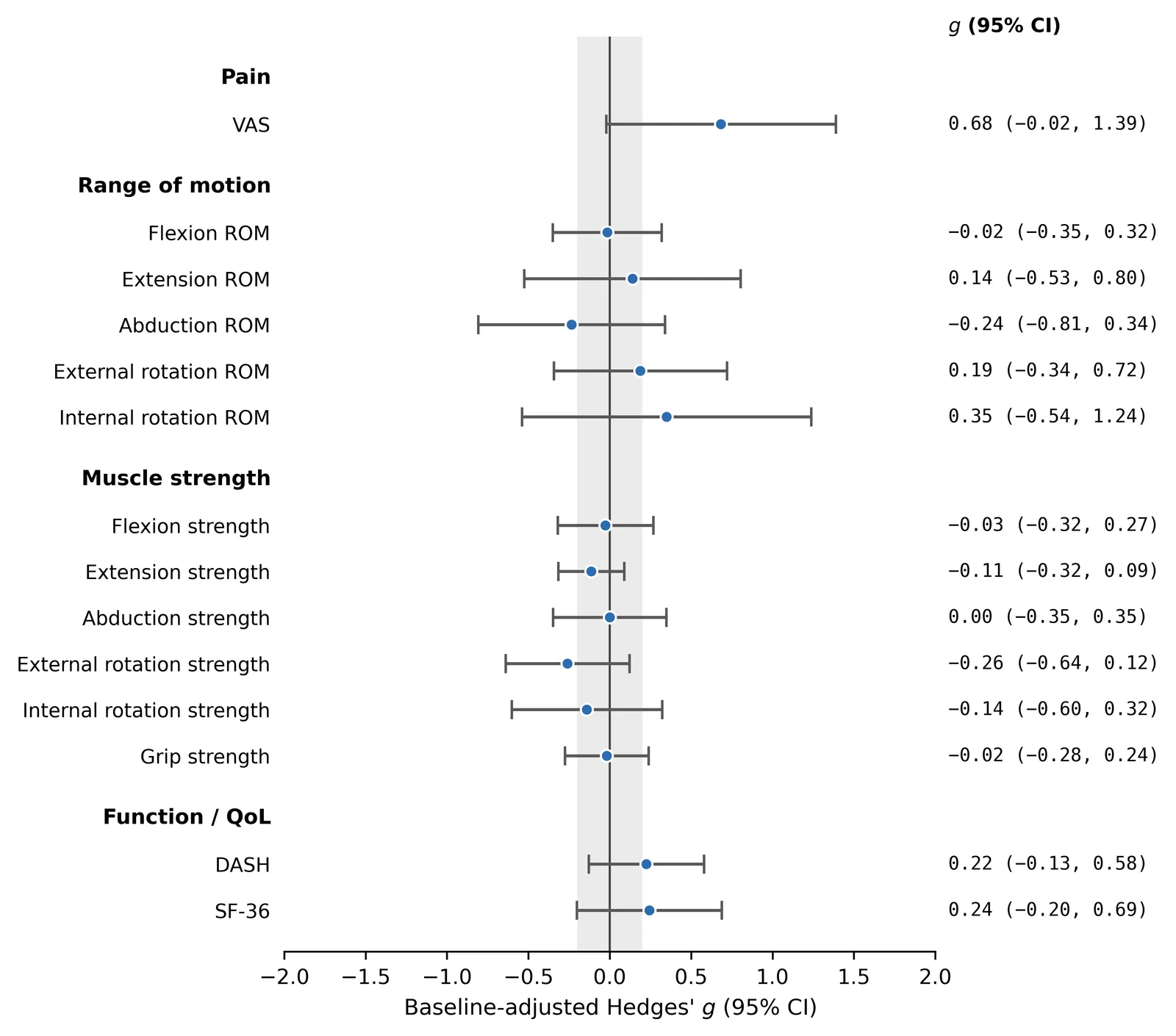
Baseline-adjusted between-group effect sizes for all 14 clinical outcomes (numerical values in Table 3). Points are Hedges’ *g* derived from analysis of covariance with the post-intervention value as the dependent variable, group as a fixed factor, and the corresponding baseline value as a covariate; horizontal lines are 95% confidence intervals. Effect sizes are oriented so that positive values favour the intervention group for every outcome, irrespective of whether higher or lower raw scores indicate improvement; consequently the sign shown for VAS and DASH is reversed relative to Table 3. The shaded band spans *g* = −0.2 to 0.2 and marks the region conventionally regarded as a negligible standardized difference. Outcomes are grouped by clinical domain. The confidence interval included the null for all 14 outcomes, and interval widths ranged from 0.4 to 1.8 standard deviation units.

Fourteen clinical outcomes were analyzed: pain intensity (VAS); five range-of-motion measures; six muscle strength measures, including grip strength; and two patient-reported measures (DASH and SF-36). Given the baseline imbalance documented in Table 1, analysis of covariance was used as the primary analytic framework for all 14 outcomes, with the post-intervention value as the dependent variable, group as a fixed factor, and the corresponding baseline value as a covariate. Adjusted between-group differences are reported in the original units of each outcome with 95% confidence intervals, and as adjusted Hedges’ *g*, calculated as the adjusted mean difference divided by the pooled baseline standard deviation with small-sample correction. Signs follow the original scale of each measure, so negative values indicate improvement for VAS and DASH and positive values indicate improvement for all other outcomes; in the forest plot (Figure 3, the estimates are re-oriented so that positive values favour the intervention group for every outcome. For each model, homogeneity of regression slopes was assessed by the group-by-covariate interaction term, influential observations by Cook’s distance, and residual normality by the Shapiro–Wilk test; these diagnostics are reported in Supplementary Table S2.

Unadjusted between-group comparisons of change scores, defined as post-intervention minus baseline, were performed as a sensitivity analysis using the Mann–Whitney *U* test. To provide an effect estimate corresponding to the rank-based comparison, the accompanying estimate was the Hodges–Lehmann median difference, with a distribution-free confidence interval. Under a location-shift model, this estimator targets the same between-group location-shift parameter to which the Mann–Whitney comparison pertains.

Because 14 outcomes were analyzed, *p* values were adjusted using the Benjamini–Hochberg procedure to control the false discovery rate, applied separately within the primary analysis and within the sensitivity analysis; both unadjusted *p* values and adjusted q values are reported. The reported *p* values are presented descriptively rather than as tests of prespecified hypotheses. All tests were two-sided. Analyses were performed in Python 3.12 (Python Software Foundation, Beaverton, OR, USA) using statsmodels, SciPy, and pandas.

## 3. Results

Thirty participants were randomized, 15 to the intervention group and 15 to the control group, and all completed the 4-week assessment. The two groups were comparable in age (56.67 ± 7.46 vs. 56.93 ± 8.41 years), height, sex distribution, and side of shoulder involvement, with absolute standardized mean differences of 0.15 or below for all four characteristics (Table 1).

Baseline imbalance was nonetheless present and was concentrated in pain intensity and passive range of motion. The intervention group reported higher baseline pain (VAS 6.13 ± 2.07 vs. 5.00 ± 1.60; SMD +0.61) and was more restricted in every rotational and elevation plane, most markedly in abduction (118.60° ± 31.24° vs. 152.73° ± 21.38°; SMD −1.28), internal rotation (39.73° ± 17.19° vs. 57.33° ± 10.50°; SMD −1.24), external rotation (SMD −0.82), and flexion (SMD −0.79). Body weight also differed (59.80 ± 7.79 vs. 65.93 ± 14.97 kg; SMD −0.51). In total, 6 of the 19 baseline characteristics exceeded an absolute standardized mean difference of 0.50.

By contrast, all six muscle strength measures, shoulder extension range of motion, and both patient-reported measures (DASH and SF-36) were closely matched, with absolute standardized mean differences not exceeding 0.19. The imbalance was therefore directional as well as substantial: the intervention group entered the trial with more severe pain and greater motion restriction, and consequently with greater scope for measured improvement, whereas the two groups did not differ appreciably in strength or self-reported function. Because change scores are sensitive to this pattern, all between-group outcome comparisons in this report use baseline-adjusted analysis of covariance, with unadjusted change scores retained only as a sensitivity analysis (Supplementary Table S1).

Engagement records were successfully retrieved for 14 of the 15 participants allocated to the intervention group (93.3%); in the remaining case the cloud-stored record could not be retrieved, although that participant completed the study and contributed to all clinical outcome analyses. Among participants with retrievable data, at least one exercise set was completed on a mean of 61.0% (SD 27.9) of the 28 available training days, with a median of 62.5% (IQR 43.8–83.0). Individual engagement varied widely, ranging from 7.1% to 96.4% of available days, corresponding to 2 and 27 recorded training days, respectively (Table 2).

Descriptive attainment thresholds, defined post hoc, showed that 12 of the 14 participants (85.7%) recorded use on at least 20% of available days, 9 (64.3%) on at least half, and 4 (28.6%) on at least 80%. These figures describe the extent to which system use could be captured in this sample. Because home-exercise participation was not recorded in the control group, they do not permit any comparison of adherence between groups.

Post-intervention values and baseline-adjusted between-group estimates for all 14 outcomes are presented in Table 3 and Figure 3. The 95% confidence interval for the adjusted effect size included the null for all 14 outcomes, and no outcome had a Benjamini–Hochberg adjusted q value below 0.05.

The largest adjusted estimate was for pain. VAS at 4 weeks was 1.30 points lower in the intervention group (95% CI −2.64 to 0.04; adjusted Hedges’ *g* = −0.68, 95% CI −1.39 to 0.02; *p* = 0.057). Among the range-of-motion outcomes, the largest adjusted effect size was for internal rotation (*g* = 0.35, 95% CI −0.54 to 1.24; adjusted mean difference 5.10°, 95% CI −7.91 to 18.12; *p* = 0.428). All six muscle strength outcomes had adjusted effect sizes of |*g*| ≤ 0.26, and the two patient-reported measures |*g*| ≤ 0.24.

Confidence interval widths for the adjusted effect sizes ranged from 0.4 standard deviation units (shoulder extension strength) to 1.8 units (internal rotation range of motion).

No adverse events were reported by participants in either group during the 4-week intervention period. No participant discontinued the intervention or withdrew from the study for any reason.

## 4. Discussion

In this pilot randomized trial, adding a gamified, asynchronously monitored home rehabilitation system to conventional outpatient rehabilitation did not produce a detectable advantage on any of the 14 clinical outcomes assessed. After adjustment for baseline values, no outcome reached nominal statistical significance, and the 95% confidence interval included the null in every case. The unadjusted change-score analysis reached nominal significance for two outcomes, pain and internal rotation, but neither survived correction for multiple comparisons, and the estimate for pain already extended to the null (Hodges–Lehmann median difference −2.0 points, 95% CI −4.0 to 0.0). Two analytic frameworks resting on different assumptions therefore converged on the same conclusion.

This convergent null result should not be read as evidence that the intervention is ineffective. Precision varied considerably across outcomes: the width of the confidence interval for the adjusted effect size ranged from 0.4 standard deviation units for shoulder extension strength to 1.8 units for internal rotation range of motion. Estimates for the muscle strength outcomes were relatively precise and centred close to the null, providing reasonable grounds to exclude a moderate or larger effect on strength within this timeframe. For pain and range of motion, however, the intervals were wide enough that the data cannot distinguish a clinically important difference from no difference at all. Notably, the two outcomes that had reached nominal significance in the unadjusted analysis, pain and internal rotation, were also the two estimated with the least precision.

Pain produced the largest adjusted estimate in the trial: 1.30 points lower on the visual analog scale at 4 weeks (95% CI −2.64 to 0.04; adjusted Hedges’ *g* = −0.68, 95% CI −1.39 to 0.02). Three considerations constrain what can be claimed from it. The estimate was unstable across analytic frameworks, being nominally significant as an unadjusted change score, borderline after adjustment, and surviving correction for multiple comparisons in neither. The intervention group also began with higher baseline pain (6.13 ± 2.07 vs. 5.00 ± 1.60; standardized mean difference +0.61), so part of the apparent advantage reflects greater scope for reduction. Most consequentially for any causal reading, concomitant analgesic use and treatments received outside the study protocol were not monitored, so a difference in co-interventions cannot be distinguished from an effect of the home programme. A mechanistic account would therefore be premature, and none is offered here. What the pain data do provide is a usable parameter for the design of a confirmatory trial: a pooled baseline standard deviation of 1.85 VAS points, from which a target sample size can be derived once a clinically meaningful difference is specified.

The apparent advantage in internal rotation is best understood as a consequence of baseline imbalance rather than as a treatment effect. At baseline, the intervention group was markedly more restricted in internal rotation than the control group (39.73° ± 17.19° vs. 57.33° ± 10.50°; standardized mean difference −1.24) and therefore had considerably greater scope for measurable gain. In the unadjusted change-score analysis, the intervention group improved by a median of 18.0° compared with 8.0° in the control group, corresponding to a Hodges–Lehmann median difference of 11.0° (95% CI 2.0 to 22.0; *p* = 0.020). After adjustment for the baseline value, the estimated difference fell to 5.10° with a confidence interval spanning 26° (95% CI −7.91 to 18.12; *p* = 0.428), and model diagnostics indicated that this instability did not arise from any single influential observation (maximum Cook’s distance 0.17). External rotation, where the intervention group also began from a substantially lower baseline, behaved in the same way, with the unadjusted advantage largely disappearing after adjustment. That both rotational measures reproduce this behaviour points to differential room for improvement rather than a directional treatment response.

System logs recorded home-training participation for 14 of the 15 participants allocated to the intervention group; in one case the cloud-stored record could not be retrieved. Across those 14 participants, at least one exercise set was completed on a mean of 61.0% (SD 27.9) of the 28 available days. This demonstrates that day-level participation can be captured automatically under low-supervision home use, addressing a measurement problem that self-report alone does not solve: existing measures of adherence to unsupervised home exercise rely predominantly on patient recall, and few have been shown to possess adequate psychometric properties [7]. It does not, however, demonstrate improved adherence. Control participants received written home-exercise instructions but no comparable record was available for them, so the trial provides no basis for comparing home-exercise participation between groups, and the engagement figure should not be read as evidence that asynchronous monitoring increases what patients actually do at home.

The distribution is more informative than the mean. Individual engagement ranged from 7.1% to 96.4%, with four participants completing at least 80% and two completing less than 20%. Variation of this magnitude has a direct implication for trial design: under intention-to-treat analysis, an effect present only among substantial users would be diluted by those who barely engage. Future trials of this delivery model will need to be powered with this heterogeneity in mind and would benefit from participation data collected on equal terms in both arms. Given the limitations of self-report, this may require providing control participants with a recording device that does not deliver the intervention.

This measurement asymmetry is not unique to the present trial. Adherence measurement across this literature has varied substantially. One app-guided feasibility study derived adherence solely from application log files, with no comparator group [18]; a randomized trial of digital versus conventional physical therapy captured engagement in both arms, but by different methods [19]. Neither measures unsupervised home exercise on equal terms, and neither does the present trial.

In adhesive capsulitis specifically, Yeo et al. evaluated an AR/Kinect-based telerehabilitation system with physician-supervised remote monitoring and found improvement over time in both groups without a significant group-by-time interaction [17]. The present trial, which used a substantially lower-supervision model, reached the same conclusion. Taken together with the mixed findings from smartphone-supported self-rehabilitation and wearable monitoring, the pattern across this small literature is one of feasible implementation without demonstrated incremental clinical benefit over conventional home exercise. Rather than testing superiority over any existing model, the present trial characterizes what a low-supervision, asynchronously monitored approach yields in a pragmatic outpatient context: effect estimates uniformly compatible with no benefit, objectively documented engagement that varies widely between individuals, and no observed harm.

Beyond the outcome estimates, the trial establishes that this delivery model can be implemented in routine outpatient care. All 30 randomized participants completed the 4-week protocol, with no withdrawals and no missing clinical outcome data. No adverse events were reported in either group, and no participant was withdrawn for inability to operate the system. These observations were not, however, evaluated against feasibility criteria specified in advance; a definitive trial should define acceptable thresholds for recruitment, retention, engagement, and data capture before enrolment begins.

Several limitations qualify these findings. The trial was retrospectively registered, and the IRB-approved protocol did not prospectively specify a primary–secondary outcome hierarchy. The outcome classification used in the registry and in this report was therefore assigned after both data collection and statistical analysis had been completed, and the possibility of data-informed outcome prioritization cannot be excluded. Accordingly, all findings, including the outcomes entered as primary in the registry, should be interpreted as exploratory and hypothesis-generating, and require confirmation in a prospectively registered, adequately powered trial.

The target sample of 30 participants reflects the pilot nature of this study, which was designed to estimate preliminary effect sizes and to characterize system feasibility rather than to establish efficacy. With 15 participants per arm, the confidence intervals for most outcomes remain compatible with effects in either direction, and the study had no capacity to detect differences in the magnitude that would be clinically relevant. Despite randomization, substantial baseline imbalance was present in pain and in four of the five range-of-motion measures, consistently favouring the intervention group; baseline pain was higher in the intervention group (standardized mean difference +0.61), and the imbalance in internal rotation was particularly marked (−1.24). This imbalance was addressed analytically through covariate adjustment, and the internal-rotation result is interpreted accordingly; adjustment cannot, however, recover the internal validity that allocation concealment would have provided.

Allocation concealment was not implemented: group assignment followed the sequence generated by a random number table, accessible to the personnel recruiting and screening participants at enrolment, without a centralized or third-party randomization procedure. Random sequence generation alone does not protect against selection bias, and the designation of this trial as randomized should not be taken to imply the usual safeguards against systematic between-group differences. Outcome assessment was additionally unblinded and was performed by the physical therapist who also delivered the physical therapy and the system training, which is of particular concern for participant-reported outcomes such as the VAS.

The diagnostic criteria for adhesive capsulitis were not operationally defined. Eligibility relied on clinical diagnosis without prespecified thresholds for the pattern or magnitude of range-of-motion restriction and without formal exclusion criteria for competing shoulder disorders; disease stage, symptom duration, imaging findings, and relevant comorbidities were likewise not systematically recorded, and none of these can be reconstructed retrospectively [20,21,22]. Adhesive capsulitis follows a stage-dependent natural course, and participants at different stages may differ both in expected trajectory and in capacity to undertake home exercise [22]. The extent of such heterogeneity in this sample therefore cannot be determined, and it may contribute to the wide variation observed in both clinical outcomes and system engagement. The possibility that some participants had co-existing or alternative shoulder pathology contributing to pain and motion loss cannot be excluded either, and this adds to the unmeasured clinical heterogeneity of the sample. This also limits reproducibility. In addition, analgesic use and treatments received outside the study protocol were neither restricted nor systematically monitored, so a difference in co-interventions between groups cannot be excluded as an explanation for the observed pain estimate.

Follow-up was limited to 4 weeks. No conclusions can be drawn regarding the durability of any effect, nor regarding outcomes over the longer natural course of adhesive capsulitis, which typically extends well beyond this interval. System-recorded engagement was available only in the intervention group and could not be retrieved for one participant owing to a cloud data retrieval failure; no comparable record of home-exercise participation was available for the control group, precluding any between-group comparison of adherence. Predefined feasibility criteria for recruitment, retention, engagement, and data capture were not specified in advance. System-generated performance metrics were not calibrated to each participant’s maximal range of motion and were not analyzed as movement-quality outcomes, and psychological factors relevant to home exercise, including pain-related fear and self-efficacy, were not measured. Finally, the trial was conducted at a single university-affiliated medical centre in Taiwan, and the findings may not generalize to other healthcare settings, rehabilitation service models, or patient populations.

Future adequately powered randomized trials should incorporate concealed allocation, blinded outcome assessment, and longer follow-up. They should also apply explicit diagnostic criteria, formal exclusion criteria for competing shoulder disorders, and disease staging at enrolment. Stratification by baseline range-of-motion severity or disease stage would reduce the risk of the imbalance encountered here and would allow for examination of whether response differs across the disease course [21,22]. Parallel participation monitoring in both arms, individually calibrated movement-quality metrics, and direct measurement of psychosocial constructs would permit evaluation of both the therapeutic dose and the mechanisms of this delivery model.

## 5. Conclusions

In this pilot randomized trial, adding a low-supervision, asynchronously monitored home rehabilitation system to conventional outpatient rehabilitation did not produce a detectable advantage on any of the 14 clinical outcomes assessed, and the confidence intervals were too wide to determine whether a clinically meaningful effect exists. The apparent advantages in pain and internal rotation in unadjusted analyses did not persist after adjustment for baseline imbalance and did not survive correction for multiple comparisons. What the trial does establish is narrower: the system could be implemented alongside routine outpatient care, all participants completed the 4-week protocol, no adverse events were reported, and home-training participation could be documented automatically, though it varied widely between individuals. Whether this delivery model confers clinical benefit remains an open question that requires a prospectively registered trial with concealed allocation, blinded outcome assessment, and adequate power.

## Figures and Tables

**Table 1:** Comparison of demographic characteristics between the control group and intervention.

Characteristic	Intervention (*n* = 15)	Control (*n* = 15)	SMD
Age (years) ^a^	56.67 ± 7.46	56.93 ± 8.41	−0.03
Height (cm) ^a^	163.53 ± 5.30	163.45 ± 9.27	+0.01
Weight (kg) ^a^	59.80 ± 7.79	65.93 ± 14.97	−0.51
VAS ^a^	6.13 ± 2.07	5.00 ± 1.60	+0.61
**Gender (** ***n*** **)**			
Male/Female **^b^**	4/11	5/10	+0.15
**Affected Side ^b^**			
Left/Right	7/8	8/7	+0.13
**Range of motion**			
**(degree) ^a^**			
Flexion	127.40 ± 25.66	147.53 ± 25.56	−0.79
Extension	35.60 ± 8.63	36.27 ± 13.02	−0.06
Abduction	118.60 ± 31.24	152.73 ± 21.38	−1.28
External rotation	29.87 ± 19.33	44.40 ± 16.15	−0.82
Internal rotation	39.73 ± 17.19	57.33 ± 10.50	−1.24
**Muscle strength ^a^**			
Flexion	10.51 ± 6.23	10.39 ± 6.36	+0.02
Extension	11.41 ± 6.11	11.74 ± 6.28	−0.05
Abduction	9.24 ± 4.13	9.43 ± 4.29	−0.05
External rotation	6.45 ± 3.27	6.67 ± 3.58	−0.07
Internal rotation	7.44 ± 4.06	7.59 ± 3.57	−0.04
Grip strength	23.93 ± 9.62	23.40 ± 9.30	+0.06
**Function and quality of life ** ^a^			
DASH	21.06 ± 8.86	22.62 ± 14.38	−0.13
SF-36	65.44 ± 18.11	61.91 ± 19.13	+0.19

Abbreviations: VAS: Visual Analog Scale; DASH: Disabilities of the Arm, Shoulder and Hand; SF-36: 36-Item Short Form Health Survey. Values are mean ± SD or n. SMD = standardized mean difference (intervention minus control). In accordance with CONSORT recommendations, no hypothesis tests were performed for baseline comparisons. An absolute standardized mean difference exceeding 0.50 was regarded as indicating imbalance of a magnitude warranting covariate adjustment in the outcome analyses. ^a^ For continuous variables, the standardized mean difference was calculated as the difference in means divided by the pooled baseline standard deviation. ^b^ For dichotomous variables, the standardized mean difference was calculated as the difference in proportions divided by the pooled standard deviation of the binomial proportions, so that these values are directly comparable with those of the continuous variables. The proportion of the second category listed (female; right) was used as the reference.

**Table 2:** Home-training engagement in the intervention group.

Measure	Intervention Group
Participants with retrievable engagement data, *n*	14/15
Engagement rate, %—mean ± SD	61.0 ± 27.9
Engagement rate, %—median [IQR] Engagement rate, %—range	62.5 [43.8, 83.0] 7.1–96.4 (2–27 of 28 days)
Participants attaining ≥20%, *n* (%)	12/14 (85.7)
Participants attaining ≥50%, *n* (%)	9/14 (64.3)
Participants attaining ≥70%, *n* (%)	7/14 (50.0)
Participants attaining ≥80%, *n* (%)	4/14 (28.6)

Engagement was defined as the number of days on which at least one exercise set was completed during the 28-day intervention period, expressed as a percentage of the 28 available training days. Values are mean ± SD or median [IQR] unless otherwise indicated. Percentages for the attainment thresholds are calculated among participants with retrievable engagement data (n = 14). Attainment thresholds were defined post hoc for descriptive purposes.

**Table 3:** Baseline-adjusted between-group treatment effects (primary analysis).

Outcome	Intervention, Post	Control, Post	Adjusted Mean Difference (95% CI)	Adjusted Hedges’ g (95% CI)	*p*	*q*
VAS	1.87 ± 1.92	2.87 ± 1.55	−1.30 (−2.64 to 0.04)	−0.68 (−1.39 to 0.02)	0.057	0.765
**Range of motion**						
Flexion	165.07 ± 12.33	168.27 ± 10.06	−0.42 (−9.22 to 8.38)	−0.02 (−0.35 to 0.32)	0.922	0.993
Extension	48.40 ± 11.65	47.20 ± 11.77	1.58 (−5.98 to 9.13)	0.14 (−0.53 to 0.80)	0.672	0.941
Abduction	151.93 ± 25.01	166.47 ± 7.17	−6.47 (−22.27 to 9.32)	−0.24 (−0.81 to 0.34)	0.408	0.831
External rotation	48.47 ± 19.38	55.80 ± 15.85	3.45 (−6.31 to 13.20)	0.19 (−0.34 to 0.72)	0.475	0.831
Internal rotation	63.13 ± 16.12	67.00 ± 16.08	5.10 (−7.91 to 18.12)	0.35 (−0.54 to 1.24)	0.428	0.831
**Muscle strength**						
Flexion	13.00 ± 5.92	13.07 ± 6.25	−0.17 (−2.07 to 1.73)	−0.03 (−0.32 to 0.27)	0.854	0.993
Extension	12.98 ± 5.76	13.99 ± 5.28	−0.73 (−2.02 to 0.56)	−0.11 (−0.32 to 0.09)	0.255	0.765
Abduction	11.45 ± 4.07	11.63 ± 4.48	0.00 (−1.52 to 1.51)	0.00 (−0.35 to 0.35)	0.996	0.996
External rotation	7.97 ± 2.58	9.06 ± 3.56	−0.92 (−2.26 to 0.42)	−0.26 (−0.64 to 0.12)	0.172	0.765
Internal rotation	9.11 ± 4.50	9.83 ± 5.51	−0.55 (−2.37 to 1.26)	−0.14 (−0.60 to 0.32)	0.536	0.834
Grip strength	26.13 ± 9.72	25.80 ± 9.59	−0.18 (−2.68 to 2.33)	−0.02 (−0.28 to 0.24)	0.885	0.993
**Function and quality of life**						
DASH	8.16 ± 4.68	11.57 ± 9.56	−2.76 (−7.11 to 1.59)	−0.22 (−0.58 to 0.13)	0.204	0.765
SF-36	74.15 ± 17.05	66.42 ± 22.02	4.65 (−3.88 to 13.19)	0.24 (−0.20 to 0.69)	0.273	0.765

Abbreviations: VAS: Visual Analog Scale; DASH: Disabilities of the Arm, Shoulder and Hand; SF-36: 36-Item Short Form Health Survey, CI: confidence interval. Values are post-intervention mean ± SD; baseline values for all outcomes are reported in Table 1. Estimates are derived from analysis of covariance with the post-intervention value as the dependent variable, group as a fixed factor, and the corresponding baseline value as a covariate. The adjusted mean difference is expressed in the original units of each outcome and represents the intervention group minus the control group. For VAS and DASH, negative values indicate improvement; for all other outcomes, positive values indicate improvement. Adjusted Hedges’ g = adjusted mean difference divided by the pooled baseline SD, with small-sample correction. q = Benjamini–Hochberg false discovery rate across all outcomes in this table. Homogeneity of regression slopes, influence diagnostics, and residual normality were examined for every model and are reported in the Supplementary Table S2. No outcome was prospectively designated as primary. All analyses are exploratory, and the *p* values are presented descriptively rather than as tests of prespecified hypotheses.

## Data Availability

The data presented in this study are available on request from the corresponding author due to privacy and ethical restrictions related to patient data. The statistical analysis code used to generate the reported results is also available from the corresponding author on request.

## Supplementary Materials

The following supporting information can be downloaded at: https://www.mdpi.com/article/10.3390/life16091551/s1, Table S1: Unadjusted between-group comparisons of change scores (sensitivity analysis); Table S2: Assumption diagnostics for the analysis of covariance models reported in Table 3; File S1: CONSORT 2025 checklist.

## Abbreviations

ANCOVA	Analysis of Covariance
AR	Augmented Reality
DASH	Disabilities of the Arm, Shoulder and Hand
SF-36	36-Item Short Form Health Survey
VAS	Visual Analog Scale
