# Supplementary material for: Low-Supervision Asynchronously Monitored Home Rehabilitation for Adhesive Capsulitis: A Pilot Randomized Study
Source: Life (Basel). 2026 Sep 16;16(9):1551. doi: 10.3390/life16091551 (PMC13608467; doi:10.3390/life16091551)
Supplement: Supplementary file 1 [file life-16-01551-s001.zip › Supplementary_Table S1.pdf]

**Supplementary Table S1. Unadjusted change-score comparison (sensitivity analysis)**

| Outcome | Intervention Δ, median [IQR] | Control Δ, median [IQR] | Hodges–Lehmann median difference (95% CI) | p | q |
|---|---|---|---|---|---|
| VAS | -4.0 [-6.0, -3.0] | -2.0 [-4.0, -1.0] | -2.00 (-4.00 to 0.00) | .026 | .183 |
| **Range of motion** | | | | | |
| Flexion | 34.0 [14.5, 58.0] | 20.0 [6.0, 30.5] | 16.00 (-2.00 to 37.00) | .093 | .325 |
| Extension | 18.0 [2.0, 22.0] | 12.0 [4.5, 15.0] | 4.00 (-8.00 to 10.00) | .442 | .687 |
| Abduction | 29.0 [9.5, 44.5] | 8.0 [-2.0, 32.0] | 18.00 (-1.00 to 39.00) | .089 | .325 |
| External rotation | 16.0 [11.0, 26.0] | 12.0 [1.5, 22.0] | 7.00 (-3.00 to 17.00) | .177 | .463 |
| Internal rotation | 18.0 [12.0, 26.0] | 8.0 [2.0, 15.5] | 11.00 (2.00 to 22.00) | .020 | .183 |
| **Muscle strength** | | | | | |
| Flexion | 2.4 [1.4, 3.8] | 2.3 [1.0, 4.2] | -0.10 (-2.35 to 1.70) | .934 | 1.000 |
| Extension | 2.1 [0.4, 3.1] | 3.4 [0.9, 3.9] | -0.80 (-2.00 to 0.64) | .245 | .491 |
| Abduction | 2.4 [1.5, 3.7] | 2.4 [0.4, 3.6] | 0.00 (-1.30 to 1.50) | 1.000 | 1.000 |
| External rotation | 1.6 [1.1, 2.3] | 2.1 [1.1, 3.6] | -0.60 (-2.40 to 0.70) | .198 | .463 |
| Internal rotation | 1.3 [0.2, 3.0] | 2.0 [0.3, 3.8] | -0.40 (-2.40 to 1.40) | .619 | .789 |
| Grip strength | 2.0 [-1.0, 4.5] | 2.0 [0.0, 5.5] | -1.00 (-3.00 to 2.00) | .676 | .789 |
| **Function and quality of life** | | | | | |
| DASH | -11.6 [-15.8, -10.0] | -10.8 [-17.9, -5.4] | -0.90 (-7.50 to 5.00) | .663 | .789 |
| SF-36 | 4.9 [1.0, 8.5] | 4.7 [-1.7, 9.3] | 2.69 (-4.04 to 10.92) | .320 | .559 |

Abbreviations: VAS, Visual Analog Scale; DASH, Disabilities of the Arm, Shoulder and Hand; SF-36, 36-Item Short Form Health Survey; CI, confidence interval.

Values are the median [IQR] of the within-participant change from baseline to post-intervention, calculated as the post-intervention value minus the baseline value. Between-group differences are reported as Hodges–Lehmann location-shift estimates with distribution-free confidence intervals. Under a location-shift model, these estimates target the same between-group location-shift estimand to which the Mann–Whitney *U* comparison pertains. Positive values of the median difference indicate a larger change in the intervention group; for VAS and DASH, in which lower scores indicate improvement, negative values therefore favour the intervention. *q* = Benjamini–Hochberg false discovery rate across the outcomes in this table. Standardized effect sizes are not reported here; baseline-adjusted effect estimates for the same outcomes are given in Table 3 and Figure 3.
