# Supplementary material for: Low-Supervision Asynchronously Monitored Home Rehabilitation for Adhesive Capsulitis: A Pilot Randomized Study
Source: Life (Basel). 2026 Sep 16;16(9):1551. doi: 10.3390/life16091551 (PMC13608467; doi:10.3390/life16091551)
Supplement: Supplementary file 1 [file life-16-01551-s001.zip › Supplementary_Table S2.pdf]

Supplementary Table S2. Assumption diagnostics for the analysis of covariance models reported in Table 3

Outcome	Group × covariate interaction p	Maximum Cook's D	Shapiro–Wilk p
VAS	.364	0.194	.061
**Range of motion**			
Flexion	.643	0.153	.372
Extension	.290	0.285	.599
Abduction	.435	0.217	.135
External rotation	.534	0.261	.095
Internal rotation	.362	0.166	.431
**Muscle strength**			
Flexion	.688	0.253	.520
Extension	.365	0.167	.056
Abduction	.657	0.216	.675
External rotation	.732	0.242	.099
Internal rotation	.231	0.335	.935
Grip strength	.977	0.154	.302
**Function and quality of life**			
DASH	.621	0.290	.015
SF-36	.241	0.184	.049

Abbreviations: VAS, Visual Analog Scale; DASH, Disabilities of the Arm, Shoulder and Hand; SF-36, 36-Item Short Form Health Survey.

Each row corresponds to the analysis of covariance model reported for that outcome in Table 3, with the post-intervention value as the dependent variable, group as a fixed factor, and the corresponding baseline value as a covariate. Homogeneity of regression slopes was assessed by adding a group-by-baseline interaction term to the model; a non-significant interaction indicates that the assumption is tenable. Cook's distance was computed for every observation and the maximum value across observations is reported, using a threshold of 1 to identify influential observations. Residual normality was assessed by the Shapiro–Wilk test. Two outcomes (DASH and SF-36) showed evidence of departure from residual normality; given the sample size and the robustness of analysis of covariance to moderate departures from normality, no transformation or alternative estimation was applied.
