# Supplementary material for: Low-Supervision Asynchronously Monitored Home Rehabilitation for Adhesive Capsulitis: A Pilot Randomized Study
Source: Life (Basel). 2026 Sep 16;16(9):1551. doi: 10.3390/life16091551 (PMC13608467; doi:10.3390/life16091551)
Supplement: Supplementary file 1 [file life-16-01551-s001.zip › CONSORT_2025_checklist_adhesive_capsulitis_final_revised.pdf]

CONSORT 2025 checklist

Low-Supervision Asynchronously Monitored Home Rehabilitation for Adhesive Capsulitis: A Pilot Randomized Study

ClinicalTrials.gov NCT07723755 (retrospectively registered, 19 July 2026); https://clinicaltrials.gov/study/NCT07723755

Section/Topic	Item No	CONSORT 2025 checklist item	Reported (page / section)
**Title and abstract**			
Title and structured abstract	1a	Identification as a randomised trial	p. 1 (Title)
	1b	Structured summary of the trial design, methods, results, and conclusions	p. 2 (Abstract)
**Open science**			
Trial registration	2	Name of trial registry, identifying number (with URL) and date of registration	p. 2 (Abstract, Trial Registration); p. 5 (Methods, para. 1)
Protocol and statistical analysis plan	3	Where the trial protocol and statistical analysis plan can be accessed	p. 5 (Methods, para. 1)
Data sharing	4	Where and how the individual de-identified participant data (including data dictionary), statistical code and any other materials can be accessed	p. 22 (Data Availability Statement)
Funding and conflicts of interest	5a	Sources of funding and other support (e.g., supply of drugs), and role of funders in the design, conduct, analysis and reporting of the trial	p. 21 (Funding). Includes disclosure of the commercial equipment rental arrangement
	5b	Financial and other conflicts of interest of the manuscript authors	p. 21 (Conflicts of Interest)
**Introduction**			
Background and rationale	6	Scientific background and rationale	pp. 3-4 (Introduction)
Objectives	7	Specific objectives related to benefits and harms	p. 4 (Introduction, final para.)
**Methods**			
Patient and public involvement	8	Details of patient or public involvement in the design, conduct and reporting of the trial	p. 5 (Methods, para. 1)
Trial design	9	Description of trial design including type of trial (e.g., parallel group, crossover), allocation ratio, and framework (e.g., superiority, equivalence, non-inferiority, exploratory)	p. 5 (Methods, para. 1)
Changes to trial protocol	10	Important changes to the trial after it commenced including any outcomes or analyses that were not prespecified, with reason	p. 5 (Methods, para. 1); p. 8 (Outcomes); p. 10 (Statistical analysis)
Trial setting	11	Settings (e.g., community, hospital) and locations (e.g., countries, sites) where the trial was conducted	p. 5 (Methods, para. 1 and para. 3)
Eligibility criteria	12a	Eligibility criteria for participants	pp. 5-6 (Methods, para. 3)
	12b	If applicable, eligibility criteria for sites and for individuals delivering the interventions (e.g., surgeons, physiotherapists)	p. 6 (Methods, para. 3)
Intervention and comparator	13	Intervention and comparator with sufficient details to allow replication. If relevant, where additional materials describing the intervention and comparator (e.g., intervention manual) can be accessed	pp. 6-7 (Methods); p. 8 (Figure 2)
Outcomes	14	Prespecified primary and secondary outcomes, including the specific measurement variable (e.g., systolic blood pressure), analysis metric (e.g., change from baseline, final value, time to event), method of aggregation (e.g., median, proportion), and time point for each outcome	p. 8 (Outcomes); p. 9-10 (Statistical analysis). No primary-secondary hierarchy was prespecified; see p. 5 and p. 8
Harms	15	How harms were defined and assessed (e.g., systematically, non-systematically)	p. 8 (Outcomes)
Sample size	16a	How sample size was determined, including all assumptions supporting the sample size calculation	p. 5 (Methods, para. 2)
	16b	Explanation of any interim analyses and stopping guidelines	p. 10 (Statistical analysis, final para.)
Randomisation: sequence generation	17a	Who generated the random allocation sequence and the method used	p. 5 (Methods, para. 1)

Section/Topic	Item No	CONSORT 2025 checklist item	Reported (page / section)
	17b	Type of randomisation and details of any restriction (e.g., stratification, blocking and block size)	p. 5 (Methods, para. 1)
Allocation concealment mechanism	18	Mechanism used to implement the random allocation sequence (e.g., central computer/telephone; sequentially numbered, opaque, sealed containers), describing any steps to conceal the sequence until interventions were assigned	p. 5 (Methods, para. 1); p. 19 (Limitations). Allocation concealment was not implemented
Implementation	19	Whether the personnel who enrolled and those who assigned participants to the interventions had access to the random allocation sequence	p. 5 (Methods, para. 1)
Blinding	20a	Who was blinded after assignment to interventions (e.g., participants, care providers, outcome assessors, data analysts)	p. 5 (Methods, para. 1); p. 9 (Statistical analysis). No blinding was performed
	20b	If blinded, how blinding was achieved and description of the similarity of interventions	Not applicable (open-label trial)
Statistical methods	21a	Statistical methods used to compare groups for primary and secondary outcomes, including harms	pp. 9-10 (Statistical analysis); harms, p. 10
	21b	Definition of who is included in each analysis (e.g., all randomised participants), and in which group	p. 9 (Statistical analysis)
	21c	How missing data were handled in the analysis	p. 10 (Statistical analysis, final para.)
	21d	Methods for any additional analyses (e.g., subgroup and sensitivity analyses), distinguishing prespecified from post hoc	p. 10 (Statistical analysis)
**Results**			
Participant flow, including flow diagram	22a	For each group, the numbers of participants who were randomly assigned, received intended intervention, and were analysed for the primary outcome	p. 7 (Figure 1); p. 9 (Statistical analysis)
	22b	For each group, losses and exclusions after randomisation, together with reasons	p. 7 (Figure 1). No losses or exclusions
Recruitment	23a	Dates defining the periods of recruitment and follow-up for outcomes of benefits and harms	p. 5 (Methods, para. 1)
	23b	If relevant, why the trial ended or was stopped	p. 5 (Methods, para. 2). Recruitment ended on reaching the planned pilot sample
Intervention and comparator delivery	24a	Intervention and comparator as they were actually administered (e.g., where appropriate, who delivered the intervention/comparator, how participants adhered, whether they were delivered as intended (fidelity))	p. 6 (Methods, para. 3); p. 9 (Statistical analysis); p. 16 (Results); Supplementary Table S2
	24b	Concomitant care received during the trial for each group	p. 6 (Methods, para. 6)
Baseline data	25	A table showing baseline demographic and clinical characteristics for each group	p. 11-12 (Table 1)
Numbers analysed, outcomes and estimation	26	For each primary and secondary outcome, by group: the number of participants included in the analysis; the number of participants with available data at the outcome time point; result for each group, and the estimated effect size and its precision (such as 95% confidence interval); for binary outcomes, presentation of both absolute and relative effect size	p. 13-14 (Table 2); p. 15 (Table 3); Supplementary Table S1. All outcomes are continuous; no binary outcomes
Harms	27	All harms or unintended events in each group	p. 16 (Results, final para. of engagement section)
Ancillary analyses	28	Any other analyses performed, including subgroup and sensitivity analyses, distinguishing pre-specified from post hoc	p. 10 (Statistical analysis); p. 16 (Results); Supplementary Table S1; Supplementary Figure S1
**Discussion**			
Interpretation	29	Interpretation consistent with results, balancing benefits and harms, and considering other relevant evidence	p. 17-20 (Discussion); p. 20 (Conclusions)
Limitations	30	Trial limitations, addressing sources of potential bias, imprecision, generalisability, and, if relevant, multiplicity of analyses	p. 19-20 (Discussion, limitations paras.); p. 20 (generalisability); p. 10 (multiplicity statement)

Notes

Page numbers refer to the submitted manuscript file (v13_clean). Section names are given alongside page numbers so that items remain traceable after typesetting.

This trial was registered retrospectively and the IRB-approved protocol did not prespecify a primary-secondary outcome hierarchy. All outcome analyses are therefore reported as exploratory, and item 14 should be read together with the disclosures on pages 5, 8 and 19.

Checklist item wording is reproduced from Hopewell S, Chan A-W, Collins GS, et al. CONSORT 2025 statement: updated guideline for reporting randomised trials. BMJ 2025;389:e081123.
